# Improved Methane Production by Photocatalytic CO_2_ Conversion over Ag/In_2_O_3_/TiO_2_ Heterojunctions

**DOI:** 10.3390/ma15030843

**Published:** 2022-01-22

**Authors:** Patricia Reñones, Fernando Fresno, Freddy E. Oropeza, Víctor A. de la Peña O’Shea

**Affiliations:** Photoactivated Processes Unit, IMDEA Energy, Avda. Ramón de la Sagra 3, 28935 Madrid, Spain; patricia.renones@csic.es (P.R.); freddy.oropeza@imdea.org (F.E.O.)

**Keywords:** photocatalysis, CO_2_, heterojunction, titanium dioxide, indium dioxide, artificial photosynthesis

## Abstract

In this work, the role of In_2_O_3_ in a heterojunction with TiO_2_ is studied as a way of increasing the photocatalytic activity for gas-phase CO_2_ reduction using water as the electron donor and UV irradiation. Depending on the nature of the employed In_2_O_3_, different behaviors appear. Thus, with the high crystallite sizes of commercial In_2_O_3_, the activity is improved with respect to TiO_2_, with modest improvements in the selectivity to methane. On the other hand, when In_2_O_3_ obtained in the laboratory, with low crystallite size, is employed, there is a further change in selectivity toward CH_4_, even if the total conversion is lower than that obtained with TiO_2_. The selectivity improvement in the heterojunctions is attributed to an enhancement in the charge transfer and separation with the presence of In_2_O_3_, more pronounced when smaller particles are used as in the case of laboratory-made In_2_O_3_, as confirmed by time-resolved fluorescence measurements. Ternary systems formed by these heterojunctions with silver nanoparticles reflect a drastic change in selectivity toward methane, confirming the role of silver as an electron collector that favors the charge transfer to the reaction medium.

## 1. Introduction

Currently, the scientific community is putting a great deal of effort into the search for clean fuels, in order to reduce the continuous CO_2_ emissions to the atmosphere and face the depletion of fossil fuels, which, together, cause the emerging energy and environmental global crises. A promising alternative to reduce the CO_2_ concentration in the atmosphere and to convert it into renewable fuels or chemicals is so-called artificial photosynthesis [1,2,3,4,5]. This process is based on the photoreduction of CO_2_ using H_2_O as an electron donor and solar light as energy source. The most studied catalyst for this reaction, similarly to other photocatalysis applications, is TiO_2_ because of its low cost, nontoxicity, (photo)chemical stability, and high activity relative to other semiconductors. However, TiO_2_ presents some disadvantages which limit its efficiency; it is only active under UV light and the photogenerated electron–hole pairs recombine with a high rate. In the last few years, researchers have been developing different strategies of modification to enhance the photocatalytic performance of TiO_2_, such as the use of dopants, cocatalysts, heterojunctions, single active sites, and bandgap engineering [6,7,8,9,10,11,12,13,14]. Among them, the formation of heterojunctions shows great potential because of its versatility and its effectiveness in reducing the recombination of electron–hole pairs, thus improving the charge separation and enhancing photocatalytic performance [9,15,16,17,18]. Furthermore, the use of heterojunctions can also enhance the light harvesting and extend the light absorption toward the visible range by means of a sensitization mechanism [16,19,20,21,22,23,24]. There are some kinds of heterojunctions, based on the use of inorganic semiconductors, which present higher activity than bare TiO_2_ in different photocatalytic applications, such as La_2_O_3_/TiO_2_, In_2_O_3_/TiO_2_, Fe_2_O_3_/TiO_2_, SnO_2_/TiO_2_, and V_2_O_5_/TiO_2,_ which promote charge separation and transfer, improve the light harvesting, or modify the surface chemistry of the system [25,26]. In addition, there is the possibility of creating three-component or ternary systems based on the union of oxides with a metal, thus allowing more efficient charge transfer and adding additional catalytically active sites [27,28,29,30,31].

In this work, the role of In_2_O_3_ in a heterojunction with TiO_2_ is studied as a way of increasing the photocatalytic activity for CO_2_ reduction [32,33,34,35,36]. This oxide was chosen due to the fact that its conduction and valence band energy levels are in proper places to be combined with TiO_2_ for promoting the migration of photoexcited electrons, which can effectively enhance the separation of electron–hole pairs and the interfacial charge transfer [37,38]. Different kinds of heterojunctions of In_2_O_3_ with commercial TiO_2_ (anatase) were synthesized while changing the particle size of the In_2_O_3_ particles, by using a commercial In_2_O_3_ and In_2_O_3_ synthesized in the laboratory, with the aim of studying the role of the size of the In_2_O_3_ particles in the properties of the catalysts and in their photocatalytic behavior [3,12,27]. Furthermore, ternary systems based on the In_2_O_3_/TiO_2_ heterojunctions and silver nanoparticles were synthesized to further enhance the charge extraction and transfer with respect to TiO_2_ and binary catalysts. Silver was selected because of its ability to improve the selectivity toward highly electron-demanding products such as methane [6,39].

## 2. Materials and Methods

### 2.1. Preparation of Catalysts

In all cases, the TiO_2_ employed was a commercial anatase-type titanium dioxide (TiO_2_, PC500) supplied by CristalACTIV^TM^ (Thann, France) In the first series of catalysts (c-series), commercial In_2_O_3_, supplied by Across Organics (Geel, Belgium), was used for the formation of the heterojunctions with TiO_2_. For the preparation of the mixed oxides, appropriate amounts of each of them for a final content of 1, 5, and 10 wt.% In_2_O_3_ were suspended in 10 mL of Milli-Q water. The suspension was then treated in an ultrasonic bath for 1 h, and the water was eliminated by evaporation at 100 °C. Afterward, the samples were ground in an agate mortar, calcined in air at 400 °C for 4 h with a temperature ramp of 10 °C/min, and finally ground again. This temperature was chosen in order to eliminate any possible organic impurity while avoiding the phase change of TiO_2_ from anatase to rutile [8,39]. The catalysts were labeled as xIn_2_O_3_-c/TiO_2_, where x indicates the nominal In amount in wt.%. For the second series (p-series), In_2_O_3_ was prepared in the laboratory using In(NO_3_)_3_·xH_2_O as a precursor, provided by Sigma-Aldrich (Darmstadt, Germany). The amount of the precursor required for the desired amount of In_2_O_3_ was dissolved in 72 mL solution of ethanol and water (3/1); then, the solvents were evaporated in a rotatory evaporator, and the obtained powder was dried at 100 °C overnight. The collected powder was calcined at 250 °C for 3 h with a temperature ramp of 10 °C/min and finally ground in an agate mortar, obtaining the sample named In_2_O_3_-p. This temperature was chosen since, according to Hoch et al. [37], it maximizes the formation of surface oxygen vacancies and hydroxyl groups, being the material most active. Then, the formation of the heterojunction between TiO_2_ and In_2_O_3_-p was performed, following the method described above for xIn_2_O_3_/TiO_2_ heterojunctions, and labeling the samples xIn_2_O_3_-p/TiO_2_, where x indicates the nominal In amount in wt.%.

Silver was incorporated by wet impregnation. The necessary amount of AgNO_3_ (Sigma Aldrich, Darmstadt, Germany) for 1 wt.% Ag was dissolved in 25 mL of Milli-Q water, and then TiO_2_ was suspended in this solution. Then, the water was eliminated in a rotatory evaporator and the powder was dried in an oven at 100 °C overnight. Afterward, the solid was ground and calcined at 400 °C for 4 h with a temperature ramp of 5 °C/min, before grinding again. Then, the formation of the heterojunction between Ag/TiO_2_ and In_2_O_3_ (In_2_O_3_-c or In_2_O_3_p) was performed, following the method described above for xIn_2_O_3_-c/TiO_2_ and xIn_2_O_3_-p/TiO_2_ heterojunctions.

### 2.2. Characterization

X-ray diffractograms were registered with a Panalytical EMPYREAN equipment (Malvern, UK) using Cu Kα radiation (λ = 1.54178 Å) with a scanning rate of 0.01°·s^−1^. The average crystal size was estimated by applying the Scherrer equation to the most intense diffraction peak of each phase. Pawley refinements were realized with the X`Pert High Score Plus software (version 2.2.1, Panalytical, Malvern, UK) for the calculation of cell parameters. Metal quantification was carried out by ICP-OES with a Perkin Elmer Optima3300 DV spectrometer (Waltham, MA, US) BET surface areas were estimated from N_2_ adsorption/desorption isotherms measured at 77 K using a QUADRASORB instrument (Quantachrome Instruments, Boynton Beach, FL, US) after degassing the samples under nitrogen at 105 °C for 20 h. Morphological properties were analyzed using a transmission electron microscope (TEM) JEOL 2100F with an energy-dispersive X-ray (EDX) detector from Oxford Instruments (Abingdon, UK) for chemical microanalysis. UV/Vis diffuse reflectance spectra were recorded in a Perkin Elmer Lambda 1050 spectrometer (Waltham, MA, USA) between 250 and 800 nm, taking BaSO_4_ as a 100% reflectance reference. Tauc plots were used to estimate optical bandgaps. Fluorescence spectra were recorded with a Perkin Elmer LS55 spectrometer (Waltham, MA, US), setting the excitation wavelength at 300 nm and filtering the emission below 350 nm. For the measurement of fluorescence lifetime, exponential decay curves were fitted to fluorescence decay data obtained by time-correlated single photon counting (TCSPC) with a Mini-τ device from Edinburgh Instruments (Livingston, UK), using as an excitation source a pulsed laser with 372 nm emission wavelength, 1 MHz pulse frequency, and 61.2 ps pulse width, and selecting a fluorescence emission of 450 ± 25 nm by means of a bandpass filter. X-ray photoelectron spectra (XPS) were taken in a SPECS spectrometer (Berlin, Germany), with an Al K_α_ X-ray source monochromated at 1486.6 eV and a PHOIBOS 150 NAP 1D-DLD analyzer. The pass energy was selected as 40 eV for survey scans and 20 eV for high-resolution scans. The binding energy scale was set using Au 4*f*_7/2_ (84.01 eV) and Ag 3*d*_5/2_ (368.20 eV). The spectra, recorded with charge compensation, were further calibrated using the C 1*s* signal of adventitious carbon. Casa XPS software (version 2.3. 24, Casa Software Ltd., Devon, UK) was used for data analysis, where Shirley or two-point linear background types were employed. Surface chemical compositions were determined using peak areas and Casa XPS sensitivity factors (C 1*s* RSF = 1.000).

### 2.3. Photocatalytic CO_2_ Reduction

Gas-phase photocatalytic CO_2_ reduction reactions were carried out in a continuous 280 mL stainless-steel reactor provided with a borosilicate glass window. Glass microfiber filters were coated with the powdered catalysts (100 mg) from a suspension in Milli-Q water and fitted into the reactor, so that the reacting gas, composed of CO_2_ (99.9999%, Praxair, Madrid, Spain) and Milli-Q water mixed in a molar ratio of 7.25 with a controlled evaporation and mixing system (CEM, Bronkorst, Ruurlo, Netherlands), flew through the filter. The reaction pressure was 2 bar, and the reaction temperature was 50 °C. Four Philips Actinic lamps (λ_max_ = 365 nm, 6 W each, Amsterdam, Netherlands) were used for UV irradiation, with a total irradiance of 50 W·m^−2^ between 330 and 400 nm, as measured with a StellarNet BLUE-Wave spectrometer (Tampa, FL, USA).

In a typical procedure, a cleaning step of 5 min in vacuum and 1 h flushing with 100 mL/min Ar were carried out. After that, an adsorption/desorption equilibration step with 30 mL/min of the reacting gas took place. Then, the reactor was pressurized, the total flow was set to 2 mL/min, and the outlet gas was analyzed in line in the dark with a gas chromatograph (Agilent 7890, MS5A, Q-PLOT and CP-Sil5B columns, two FID and one TCD detectors, Santa Clara, CA, US). One hour later, the UV source was switched on, and the reaction was allowed to proceed for 15 h. Results are expressed as the total amount of product obtained after 15 h, while C-selectivity (carbon selectivity) to methane is defined as the amount of methane produced divided by the total amount of carbon-containing products.

## 3. Results and Discussion

### 3.1. Materials Characterization

Table 1 collects the main physicochemical characteristics of the prepared catalysts. Chemical analyses by ICP-OES revealed that the indium and silver concentrations in the heterojunctions were in the range of the nominal ones. Further surface chemical analyses based on XPS also revealed a close agreement with the nominal concentration of the samples, thus confirming a homogeneous distribution of the In_2_O_3_–TiO_2_ heterojunction.

**Figure 1:**
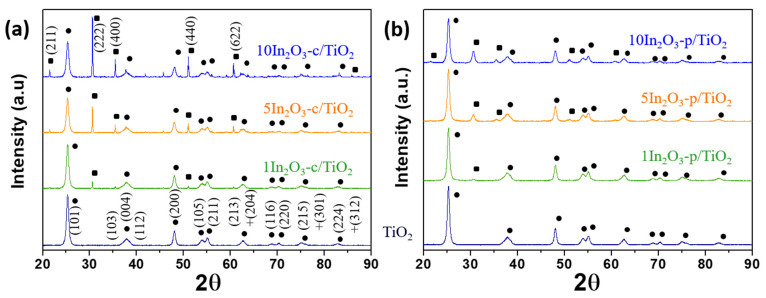
X-ray diffractograms of (**a**) c-series and (**b**) p-series catalysts. The identified phases are differentiated with symbols: ●TiO_2_ (ICCD-PDF: 01-084-1286) and ■ In_2_O_3_ (ICCD-PDF: 01-071-2195), and their Miller indices are included.

XRD diffraction patterns (Figure 1) exhibited in both series the characteristic diffraction peaks of In_2_O_3_ (ICDD-PDF: 01-071-2195) and anatase TiO_2_ (ICDD-PDF: 01-084-1286) as the only crystal phases. Pawley refinements were carried out to compare the TiO_2_ lattice cell parameters with those of bare TiO_2_ (Table 1). The calculated cell parameters of all In_2_O_3_-loaded TiO_2_ samples were in agreement with those of bare TiO_2_, corroborating the formation of a composite material instead of a possible doping. The crystal sizes, determined by the Scherrer equation (Table 1), were higher for commercial In_2_O_3_ than for In_2_O_3_-p particles, around 80 and 14 nm, respectively. In the case of TiO_2_ particles, the anatase crystal size (not shown) presented only small, nonsignificant variations in all heterojunctions with respect to bare titania. For ternary catalysts, the In_2_O_3_ and TiO_2_ phases were observed, but there were no signs of metallic silver or silver oxide phases (see Supplementary Material, Figure S1). This can be traced back to the crystal size and/or total amount of Ag phases lower than the detection limits of the technique. XPS spectra of Ag-loaded samples in the Ag 3*d* region (Figure S2), in turn, could be fitted with a pair of symmetric Voigt functions at 368.6 eV and 374.6 eV. The symmetry of the peaks and the absence of satellite peaks (characteristic for metallic Ag) indicate that silver was in the oxide state. Although as-prepared samples contained Ag oxides, such species underwent reduction under reaction conditions, leading to metallic Ag as the actual cocatalyst for the CO_2_ reduction [6,39]. According to ICP and XPS chemical analyses (see results in Table 1), the Ag surface fraction was 3.1 and 2.3 wt.% for Ag/1In_2_O_3_-c/TiO_2_ and Ag/1In_2_O_3_-p/TiO_2_, respectively. Provided that silver was only decorating the TiO_2_ surface, the higher Ag concentration in the c-series catalyst may have resulted from lower surface interaction with In_2_O_3_ particles due to the higher crystallite size of the commercial In_2_O_3_ sample [6,8,39].

The Raman analysis of all materials showed the signals of TiO_2_ anatase (Figure S3) (143 (E_g_), 196 (E_g_), 396 (B_1g_), 516 (A_1g_ + B_1g_), and 639 cm^−1^ (E_g_)) [40,41], while no signals corresponding to In_2_O_3_ (133 (E_2g_), 303 (E_1g_), 336 (E_2g_), 495 (A_1g_), and 629 cm^−1^ (E_2g_)) [42,43] were identified. Only a small shift in the most intense peak of anatase (143 cm^−1^, E_g_) [37,44] was observed, which could be related to the presence of the most intense peak of In_2_O_3_, as observed in the individual Raman spectra of In_2_O_3_ samples in Figure S2c.

Regarding textural properties (Table 1), for the p-series, a reduction in surface area occurred with the inclusion of indium oxide compared to TiO_2_, and this became more significant with the growth of In_2_O_3_ percentage. This could be related to the small size of In_2_O_3_-p particles that can enter the interparticle pores of TiO_2_. On the other hand, the c-series catalysts, which contain larger In_2_O_3_ crystallites, showed a slight increase in the surface area compared to TiO_2_. This probably occurred due to the higher crystal size of In_2_O_3_ particles, whereby they could not enter the interparticle pores of TiO_2_, avoiding the agglomeration of the particles. In all ternary systems, the area was decreased with respect to unmodified TiO_2_ and to the corresponding binary systems, which could be ascribed to partial obstruction of TiO_2_ pores by silver nanoparticles (Table 1) [6,39].

**Figure 2:**
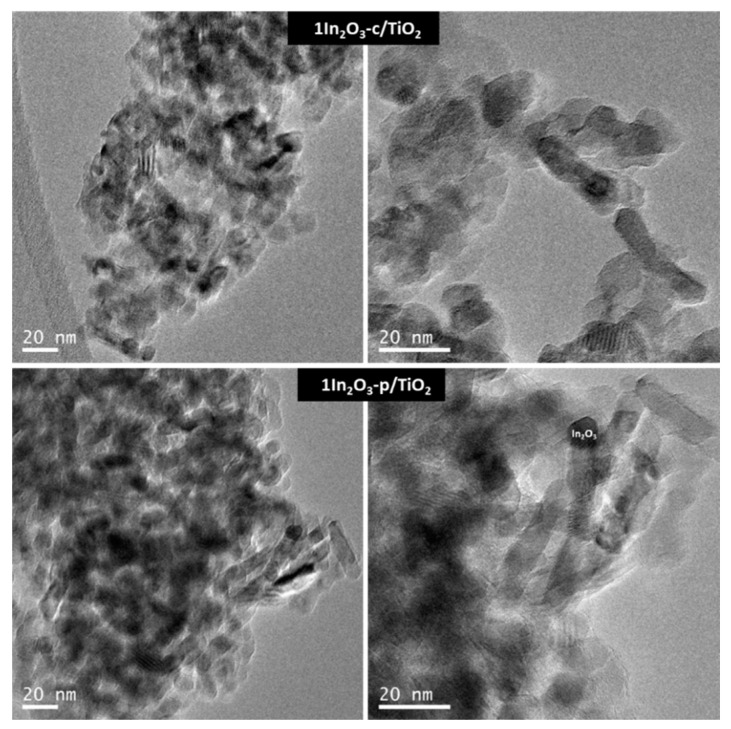
TEM micrographs of 1In_2_O_3_-c/TiO_2_ and 1In_2_O_3_-p/TiO_2_ catalysts.

Figure 2 shows the TEM images of 1% In catalysts in both series. In both cases, the TEM analysis showed a good dispersion of the In_2_O_3_. EDX analysis confirmed that the particles with darker contrast corresponded to In_2_O_3_. Furthermore, the amounts of In corresponded well with the nominal value in all analyzed samples (Figure S4).

**Figure 3:**
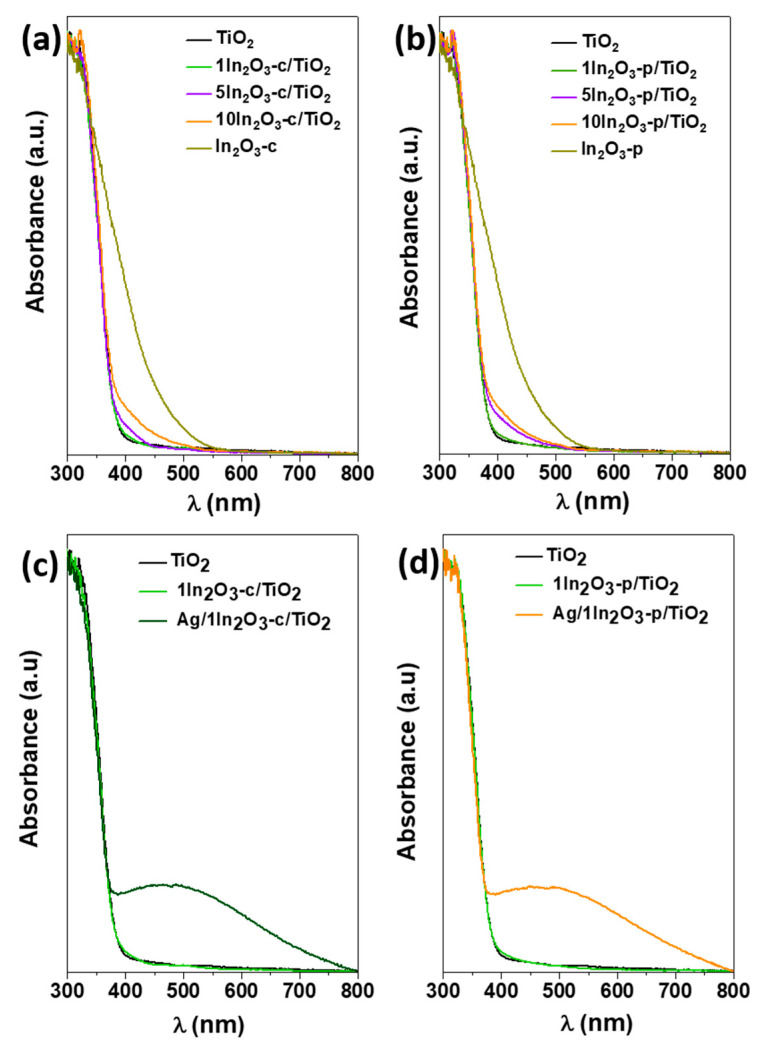
UV/Vis diffuse reflectance spectra of all catalysts studied compared to TiO_2_: (**a**) c-series, (**b**) p-series, and (**c**,**d**) ternary catalysts compared to their binary counterparts.

UV/Vis diffuse reflectance spectra (Figure 3) show that, in both c- and p-series, the presence of indium oxide led to increased absorption in the visible range, which increased with the In_2_O_3_ content and could be associated with the bandgap transition of indium oxide (2.6 eV for both commercial and synthesized In_2_O_3_). Deconvolution of TiO_2_ and In_2_O_3_ contributed to the spectra as an optical bandgap for the former of 3.1 eV in all doped samples, the same values as in bare TiO_2_, in agreement with the deduced formation of a heterojunction rather than doping. In the case of ternary catalysts, which were UV-irradiated before acquiring the spectra, the absorption generated by the surface plasmon resonance of silver particles was also observed.

**Figure 4:**
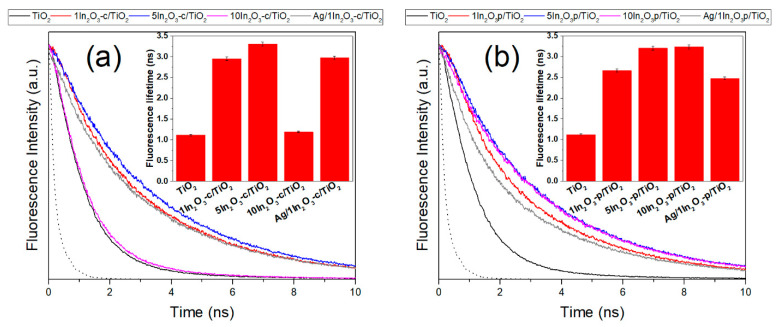
Fluorescence decay curves (main graphs) and fluorescence lifetimes obtained from fittings (insets), for the c-series (**a**) and the p-series (**b**) catalysts. Dotted lines represent the instrument response function.

Steady-state fluorescence spectra (Figure S5) show that emission wavelengths essentially matched those of TiO_2_ in all cases, indicating that the photoluminescence contribution of In_2_O_3_ was minimal, as could be expected from the low concentration of In_2_O_3_ in the studied samples. However, a decrease in the emission in comparison with bare TiO_2_ was observed, which may indicate a reduction in the recombination rate of electrons and holes [21,45,46,47] and, therefore, a charge transfer between phases. To confirm this, fluorescence lifetime measurements were carried out (Figure 4). Time-resolved spectra revealed an increased fluorescence lifetime in heterojunctions associated with electron transfer from In_2_O_3_ to TiO_2_, according to their relative band positions, with the conduction band of the indium oxide at higher energy that that of titania [37,38], such that electrons could migrate from the former to the latter; thus, the duration of the fluorescence emission increased [48]. In the c-series, this transfer increased from 0 to 1 to 5 wt.% In_2_O_3_, but decreased with 10 wt.%, suggesting that the contact between both phases was no longer efficient with high In_2_O_3_ amount. In the p-series, however, the transfer continued being efficient up to 10 wt.%, which could be traced back to an improved phase contact due to the smaller size of In_2_O_3_ crystallites [22,28,46]. The case of silver-containing catalysts was more complex, as results revealed opposite effects. On the one hand, as described above, In_2_O_3_ transferred electrons to the conduction band of titania, increasing fluorescence lifetime; on the other hand, silver withdrew charge from the TiO_2_ conduction band [6], decreasing lifetime. As a result, the value obtained for Ag/1In_2_O_3_/TiO_2_, whatever the series, was similar to or slightly lower than in 1In_2_O_3_/TiO_2_.

### 3.2. Photocatalytic Tests

**Figure 5:**
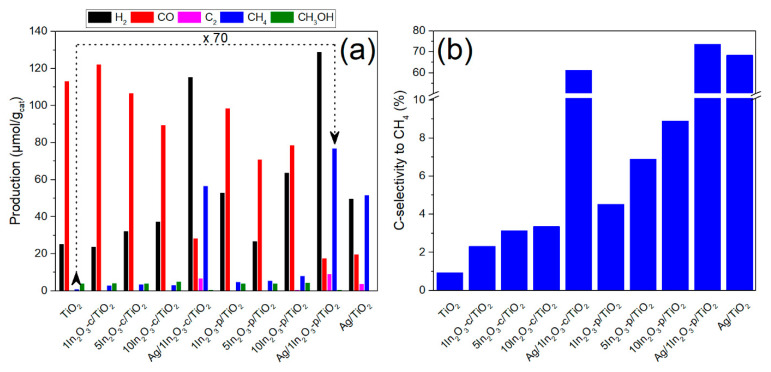
(**a**) Cumulative production of the main products obtained with different catalysts in the CO_2_ + H_2_O reaction after 15 h under UV light. (**b**) C-selectivities (%) toward CH_4_ in the same reaction.

Figure 5 represents, in the left panel, the amounts of the different products obtained in CO_2_ photoreduction over the studied catalysts after 15 h of irradiation. Bare TiO_2_ gave rise to CO as the main product, with minor amounts of CH_4_, CH_3_OH, and C_2_ (ethylene and ethane), together with hydrogen resulting from the parallel reduction of water. The incorporation of In_2_O_3_ led to changes in the product distribution with an increase in CH_4_ and H_2_ production in both In_2_O_3_/TiO_2_ series, which was higher for larger amounts of In loading. This enhancement was higher in the case of the p-series, with a more than sevenfold increase in produced CH_4_ for the 10In_2_O_3_-p/TiO_2_ catalyst with respect to TiO_2_. These changes were also combined with a slight increase in C_2_ and a decrease in CO production. Regarding In_2_O_3_, the obtained methane production was ca. 46%, obtained with TiO_2_. Therefore, improved methane production was in all cases higher than the linear combinations of the activities of the single components, revealing a synergistic effect.

This change in product distribution was previously observed and attributed to a decrease in the electron–hole recombination rate that favors the formation of highly electron-demanding products [1,49,50]. Among these products, selectivity to methane is most affected by the catalyst nature, while that to methanol and C_2_ is essentially maintained upon introduction of indium oxide. Focusing, therefore, on methane selectivity, the right panel of Figure 5 shows the values obtained with the different catalysts, considering only the carbon products as indicated in the experimental section. The graph allows observing a correlation between selectivity to methane and fluorescence lifetime (and, therefore, inter-phase charge transfer). Thus, in good accordance with the results shown in Figure 4, CH_4_ selectivity increased in the c-series, from TiO_2_ to 1In_2_O_3_-c/TiO_2_ and from this to 5In_2_O_3_-c/TiO_2_, and then the improvement was practically lost when using 10 wt.% In_2_O_3_. On the contrary, in the p-series, the selectivity was also considerably higher when introducing 1% In_2_O_3_, before further increasing with 5%; however, there was a further improvement when the amount of indium oxide was increased to 10%, which could be traced back to the maintained charge transfer observed in time-resolved fluorescence measurements. Therefore, a direct effect of this charge transfer on the selectivity toward a highly electron-demanding product such as methane could be envisaged, and this effect was more pronounced with more extensive phase contact derived from smaller In_2_O_3_ crystallites.

The effect of silver deposition on the reactivity and selectivity was studied with the 1% In_2_O_3_ samples in both c- and p-series. A great improvement in the selectivity toward CH_4_ was observed in both series, being again particularly significant for the p-series sample, which improved the production of methane attained with TiO_2_, 1In_2_O_3_-p/TiO_2_, and the previously reported [6] Ag/TiO_2_ by 70, 15.5, and 1.5 times, respectively. This improved reactivity to methane was attributed to the electron-scavenging ability of Ag nanoparticles, which further increased electron ability for intensive CO_2_ reduction into the eight-electron product CH_4_ [6].

Lastly, it is worth noting that selectivity to hydrogen against carbon products, as deduced from Figure 5 (left), evolved in a similar way as that to methane across the different indium amounts and even with the introduction of the silver cocatalyst, subtracting photoexcited electrons from being used for CO_2_ reduction. A further challenge with the present catalysts is, therefore, to drive the competition for conduction band electrons toward CO_2_, thus pursuing total selectivity to methane.

## 4. Conclusions

With the heterojunctions based on In_2_O_3_ and TiO_2_, better activities were obtained with respect to bare anatase TiO_2_. This enhancement was reflected mostly in the selectivity to methane and was related to a decreased electron–hole recombination as confirmed by fluorescence analysis. The activity results also revealed a significant change depending on the crystal size of the In_2_O_3_ employed. The smaller crystallite size of indium particles obtained in the laboratory favored methane production, but gave a lower overall conversion than the bare TiO_2_ and the heterojunctions formed between TiO_2_ and commercial In_2_O_3_, suggesting that electrons were directed toward the eight-electron reduction product CH_4_. Time-resolved fluorescence measurements allowed relating the improved methane selectivity to the transfer of photoexcited electrons from In_2_O_3_ to TiO_2_, which was more efficient with smaller In_2_O_3_ catalysts. The ternary systems formed between Ag and In_2_O_3_/TiO_2_ enabled a further increase in CH_4_ production, with the ternary catalysts prepared with synthetic In_2_O_3_ again being more active than those with commercial In_2_O_3_. As a result, the best Ag/In_2_O_3_ system improved both CH_4_ production and selectivity compared to the previously studied Ag/TiO_2_ system, and it enhanced CH_4_ production with respect to TiO_2_ by a factor of 70.

## Figures and Tables

**Table 1:** Main physicochemical characteristics of the studied catalysts.

Catalyst	Bulk In (wt.%) ^a^	Surface In (wt.%) ^b^	Bulk Ag (wt.%) ^a^	Surface Ag (wt.%) ^b^	S_BET_ (m^2^/g)	In_2_O_3_ Crystallite Size (nm)	TiO_2_ Cell Parameters (Å)	
							a = b	c
**TiO_2_**	-	-	-	-	112	-	3.7844	9.5088
**1In_2_O_3_-c/TiO_2_**	1.11 ± 0.06	1.3	-	-	121	105	3.7836	9.5072
**5In_2_O_3_-c/TiO_2_**	4.6 ± 0.2	4.0	-	-	119	68	3.7839	9.508
**10In_2_O_3_-c/TiO_2_**	9.1 ± 0.5	9.5	-	-	115	83	3.7826	9.5094
**1In_2_O_3_-p/TiO_2_**	0.89 ± 0.04	0.7	-	-	101	13	3.7811	9.5082
**5In_2_O_3_-p/TiO_2_**	4.0 ± 0.2	n.m.	-	-	104	15	3.7849	9.5106
**10In_2_O_3_-p/TiO_2_**	9.4 ± 0.4	10.5	-	-	100	16	3.7838	9.508
**Ag/1In_2_O_3_-c/TiO_2_**	0.73 ± 0.04	n.m.	0.76 ± 0.04	3.1	109	78	3.7839	9.5079
**Ag/1In_2_O_3_-p/TiO_2_**	0.73 ± 0.04	n.m.	0.79 ± 0.04	2.3	90	16	3.7841	9.5076

^a^ From ICP-OES. ^b^ From XPS in the Ti 2*p* and In 3*d* regions and respective sensitivity factors. n.m.: not measured.

## Data Availability

Not applicable.

## Supplementary Materials

The following are available online at https://www.mdpi.com/article/10.3390/ma15030843/s1: Figure S1. X-ray diffractograms of the ternary catalysts; Figure S2. XPS in the Ag 3*d* region of Ag/1In_2_O_3_-p/TiO_2_ and Ag/1In_2_O_3_-c/TiO_2_; Figure S3. Raman spectra of (a) c-series, (b) p-series, (c) In_2_O_3_-c and In_2_O_3_-p, and (d) ternary photocatalysts, compared to TiO_2_; Figure S4. EDX analysis by TEM of 1In_2_O_3_-c/TiO_2_ and 1In_2_O_3_-p/TiO_2_ photocatalysts; Figure S5. Fluorescence spectra of all catalysts.
