# Supplementary material for: Improved Methane Production by Photocatalytic CO2 Conversion over Ag/In2O3/TiO2 Heterojunctions
Source: Materials (Basel). 2022 Jan 22;15(3):843. doi: 10.3390/ma15030843 (PMC8837040; doi:10.3390/ma15030843)
Supplement: Supplementary file 1 [file materials-15-00843-s001.zip › materials-1554359-supplementary.pdf]

## Supplementary Material

# Improved methane production by photocatalytic $CO_2$ conversion over $Ag/In_2O_3/TiO_2$ heterojunctions

**Patricia Reñones, Fernando Fresno*, Freddy E. Oropeza and Víctor A. de la Peña O'Shea***

Photoactivated Processes Unit, IMDEA Energy, Avda. Ramón de la Sagra 3, 28935, Móstoles, Madrid, Spain.

**SUPPORTING INFORMATION CONTENTS**

Figure S1. X-ray diffractograms of the ternary catalysts. ................................................ 3

Figure S2. XPS in the Ag 3d region of a) Ag/1$In_2O_3$-cTi$O_2$ and Ag/1$In_2O_3$-p/Ti$O_2$......... 4

Figure S3. Raman spectra of: a) c-series, b) p-series, c) $In_2O_3$ and $In_2O_3$p and d) ternary photocatalysts, compared to $TiO_2$. ........................................................................ 5

Figure S4. EDX analysis by TEM of 1$In_2O_3$-c/$TiO_2$ and 1$In_2O_3$-p/$TiO_2$ photocatalysts. . 6

Figure S5. Fluorescence spectra of all catalysts compared to $TiO_2$. ................................ 7

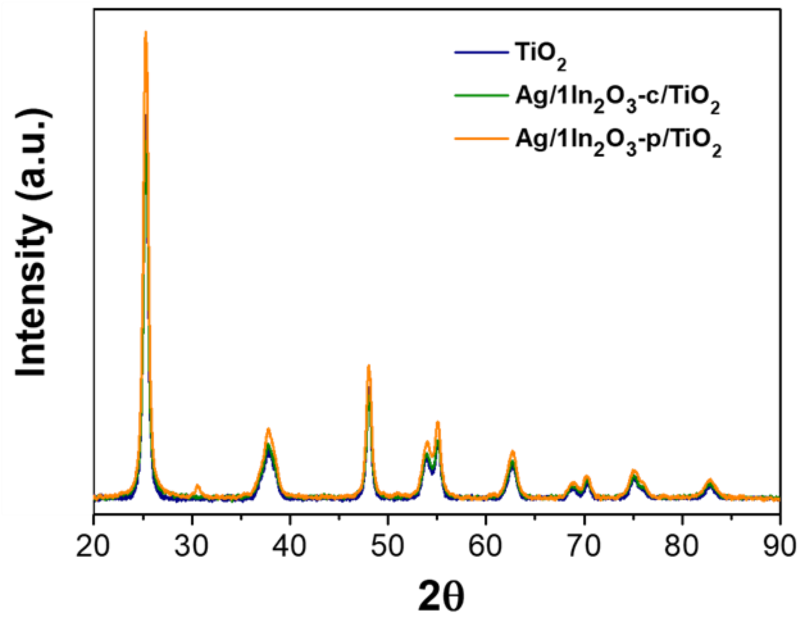


**Figure S1.** X-ray diffractograms of the ternary catalysts.

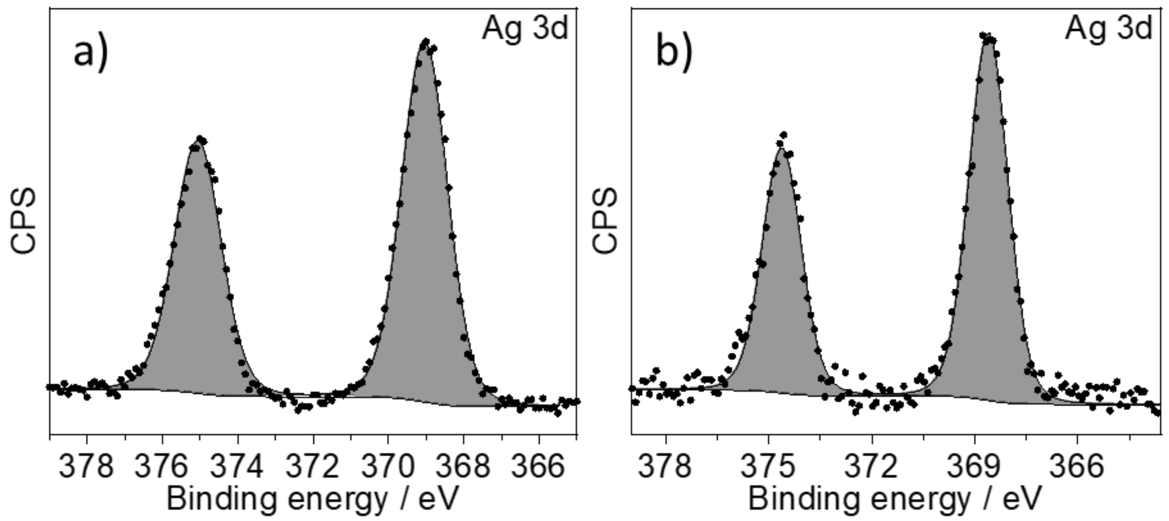


**Figure S2.** XPS in the Ag 3d region of a) Ag/1$In_2O_3$-c$TiO_2$ and Ag/1$In_2O_3$-p/$TiO_2$

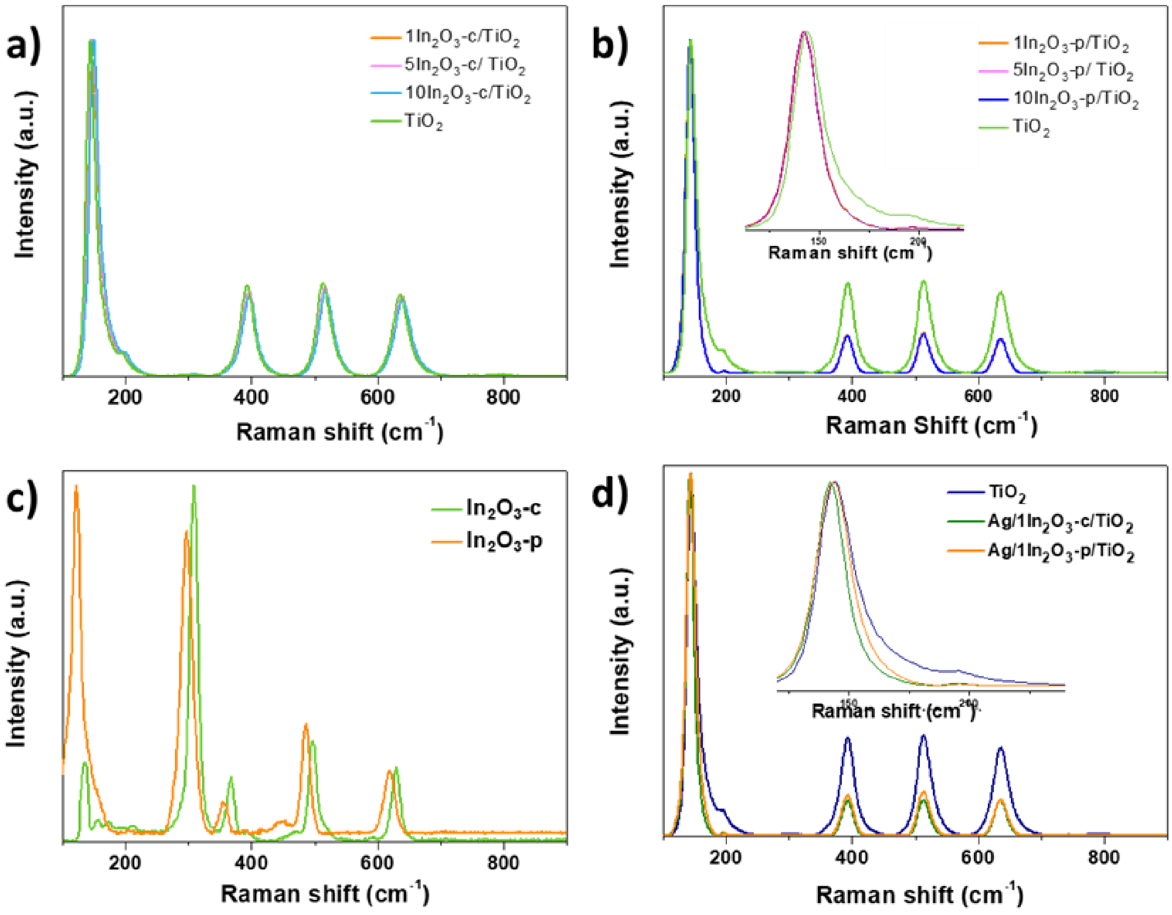


**Figure S3.** Raman spectra of: a) c-series, b) p-series, c) $In_2O_3$ and $In_2O_3$p and d) ternary photocatalysts, compared to $TiO_2$.

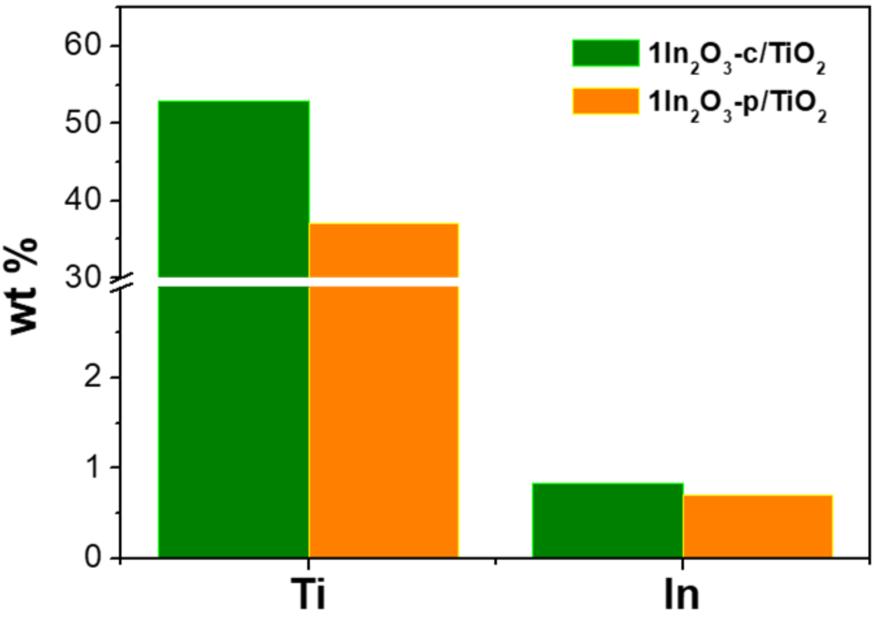


**Figure S4.** EDX analysis by TEM of 1$In_2O_3$-c/$TiO_2$ and 1$In_2O_3$-p/$TiO_2$ photocatalysts.

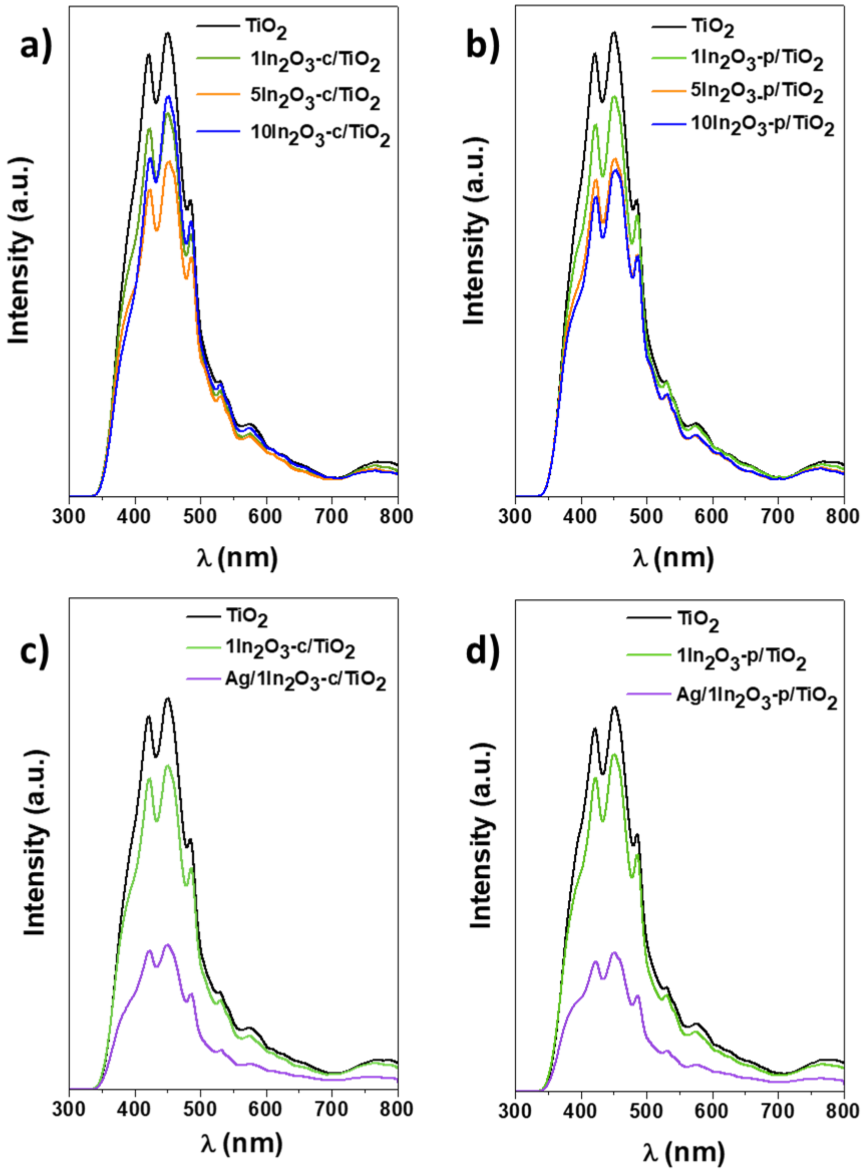


**Figure S5.** Fluorescence spectra of all catalysts compared to $TiO_2$.
